# Lacunar Pontine Infarct Presenting as Ipsilateral Lower Motor Neuron Facial Palsy: A Case Report

**DOI:** 10.7759/cureus.104930

**Published:** 2026-03-09

**Authors:** Naman Popat, Margi D Patel, Varoon J. Vadodaria

**Affiliations:** 1 Medicine, Sumandeep Vidyapeeth (Deemed to be University), Vadodara, IND; 2 Neurology, Sumandeep Vidyapeeth (Deemed to be University), Vadodara, IND

**Keywords:** aspirin, atorvastatin, clopidogrel, diabetes, facial palsy, hypertension, stroke

## Abstract

Most cases of unilateral facial paralysis are caused by Bell’s palsy, which is usually an idiopathic lower motor neuron facial nerve palsy. However, some cases result from an infarction of the ipsilateral facial nerve region in the pons, which mimics Bell’s palsy and is referred to as a pontine stroke. This study reports a case of a 63-year-old male who presented with complaints of dizziness, giddiness, nausea, vomiting, and headache for 12 hours. He developed facial deviation to the right and slurred speech seven hours before presentation. Additionally, he had a history of hypertension and diabetes. No signs of meningeal irritation were noted. Cranial nerve examination and MRI were suggestive of a left lacunar pontine infarct. The patient was given aspirin, clopidogrel, and atorvastatin along with antihypertensive medications and other symptomatic treatments. He was discharged after seven days in a stable condition. This report highlights the importance of clinicians being aware of the need to investigate for stroke when a patient presents with peripheral facial nerve palsy.

## Introduction

Unilateral facial palsy is a common neurological presentation, with Bell’s palsy accounting for approximately 60-70% of cases worldwide [1]. Bell’s palsy is characterized by an acute, idiopathic lower motor neuron paralysis of the facial nerve and typically has a favorable prognosis. However, other etiologies, including central nervous system causes such as pontine infarction, contribute to a small but clinically significant proportion of cases, with pontine stroke reported in about 1% of all facial paralysis presentations [2]. A pontine infarct involving the facial nerve nucleus or its fascicles can produce a lower motor neuron facial palsy that closely mimics Bell’s palsy, thereby creating diagnostic challenges in the acute setting. An abrupt onset, subtle brainstem symptoms such as dizziness or dysarthria, vascular risk factors, and the absence of typical Bell’s palsy features, including retroauricular pain, taste disturbance, or hyperacusis, may suggest a central etiology and warrant urgent neuroimaging.

Unlike many previously reported cases, our patient presented with near-isolated lower motor neuron facial palsy and only minimal accompanying neurological signs, closely resembling Bell’s palsy clinically. Early detection on diffusion-weighted MRI of a small dorsal pontine infarct at the facial colliculus highlights the importance of considering stroke in patients with peripheral appearing facial weakness to avoid diagnostic delay. These strokes typically result from occlusion of the paramedian perforating arteries branching from the basilar artery [3,4], while the facial nerve nucleus itself is primarily supplied by the anterior inferior cerebellar artery, another branch of the basilar artery [5]. The overlap in clinical features poses a risk of misdiagnosis, particularly in patients who lack additional brainstem signs. Timely differentiation between Bell’s palsy and pontine stroke is critical, as the management pathways and prognoses differ substantially.

While Bell’s palsy is treated with corticosteroids and supportive care, pontine infarction requires acute stroke protocols and secondary prevention to minimize neurological morbidity. Yet, despite this diagnostic importance, literature reports of isolated facial palsy due to pontine stroke remain scarce, with only a handful of cases documented globally [6-10]. In this context, we present a case of a 63-year-old male with a left lacunar pontine infarct presenting as an isolated lower motor neuron facial palsy to highlight the diagnostic challenge posed by brainstem strokes that clinically mimic Bell’s palsy, and emphasize the importance of recognizing subtle clinical red flags and performing early MRI evaluation to avoid misdiagnosis and delays in acute stroke management.

## Case presentation

A 63-year-old male, known to have hypertension and type 2 diabetes mellitus, presented to the emergency department with the chief complaint of acute facial deviation and slurred speech, accompanied by dizziness, giddiness, nausea, vomiting, and a dull, diffuse headache lasting 12 hours. These associated symptoms were atypical for Bell’s palsy and raised the possibility of an alternative central neurological cause, prompting further stroke-focused evaluation. There was no history of seizures, trauma, or prior cerebrovascular events. At presentation, the combination of acute-onset facial weakness with associated neurological symptoms and underlying vascular risk factors raised concern for a possible central neurological event rather than an isolated peripheral facial nerve palsy. Therefore, a detailed neurological examination was performed to differentiate between peripheral and central causes.

On physical examination, the patient was cooperative, alert, and oriented to time, place, and person. His vital signs showed a blood pressure of 185/80 mmHg, with normal oxygen saturation and respiratory rate. Neurological examination revealed normal higher mental functions, motor tone, muscle strength, sensory modalities, cerebellar testing, and gait assessment. No signs of meningeal irritation were elicited. Cranial nerve examination demonstrated left-sided facial weakness characterized by drooping of the left eyelid, inability to close the left eye against resistance, reduced left nasolabial fold, and downward deviation of the left angle of the mouth while smiling, consistent with a left lower motor neuron facial palsy.

Given the acute onset, the presence of vascular comorbidities, and subtle accompanying symptoms such as dizziness and slurred speech, an MRI of the brain using an acute stroke protocol, including diffusion-weighted imaging (DWI), apparent diffusion coefficient (ADC), T2-weighted, and FLAIR sequences, was performed to exclude a brainstem infarct. The scan was obtained approximately 12 hours after the onset of initial symptoms and about seven hours after the development of facial deviation and slurred speech. As the patient presented beyond the thrombolysis window and remained neurologically stable, antiplatelet therapy and secondary stroke prevention measures were initiated.

**Figure 1:**
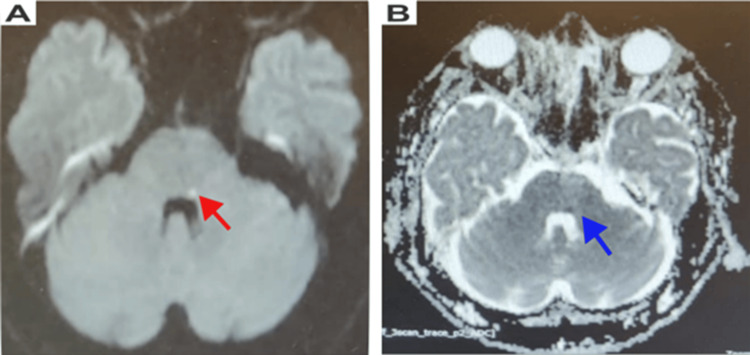
(A) MRI shows increased focus on DWI and (B) ADC restriction sequences in the dorsal aspect of the left pons at the floor of the fourth ventricle, corresponding to the left facial colliculus The small hyperintense spot in the DWI image is indicated by a red arrow, and the hypointense signal in the ADC image is shown by a blue arrow MRI: magnetic resonance imaging; DWI: diffusion-weighted imaging; ADC: apparent diffusion coefficient

Although an internal auditory canal (IAC) protocol MRI is often considered in cases of isolated peripheral facial paralysis, the acute onset of symptoms, associated brainstem features, and significant vascular risk factors in this patient favored initial evaluation with a stroke protocol MRI to rapidly exclude a central ischemic cause. The MRI revealed a small hyperintense lesion on DWI in the dorsal aspect of the left pons at the floor of the fourth ventricle, corresponding to the facial colliculus region (Figures 1A, 1B).

The imaging findings correlate with the clinical presentation of isolated lower motor neuron facial palsy and support a nuclear/fascicular pontine lesion rather than peripheral facial nerve pathology.

The lesion showed hypointensity on the ADC map, confirming the diagnosis of an acute infarct. Differential diagnoses considered included Bell’s palsy, vestibular disorders, demyelinating lesions, neoplastic processes, and inflammatory or infective causes of facial nerve palsy. Bell’s palsy was considered less likely due to the acute onset with associated brainstem-related symptoms such as dizziness and slurred speech, along with significant vascular risk factors. Vestibular and other peripheral causes were excluded based on the absence of isolated vertigo, hearing loss, or positional triggers. Furthermore, the presence of diffusion restriction with corresponding ADC hypointensity localized to the dorsal pons supported the diagnosis of an acute ischemic pontine infarct and effectively ruled out alternative etiologies. T2-weighted and FLAIR sequences showed subtle signal changes, with no additional areas of restricted diffusion identified within the brainstem. The cisternal, canalicular, and intratemporal segments of the facial nerve were carefully evaluated and showed no abnormal enhancement or signal changes, further supporting a central rather than peripheral etiology.

Laboratory investigations revealed a fasting blood glucose of 168 mg/dL and an HbA1c of 7.6%, indicating suboptimal glycemic control. The lipid profile showed a total cholesterol level of 214 mg/dL and an LDL cholesterol level of 138 mg/dL, consistent with moderate dyslipidemia. Other routine laboratory parameters, including complete blood count, renal and liver function tests, were within normal limits (Table 1). The patient was managed with aspirin 75 mg once daily, clopidogrel 75 mg once daily, and atorvastatin 40 mg once daily, along with antihypertensive medications and supportive care. His hospital course was uneventful, and he was discharged in stable condition after seven days with advice for outpatient follow-up and secondary stroke prevention measures.

**Table 1:** Laboratory investigation results RBC: red blood cell; SGPT: alanine aminotransferase; SGOT: aspartate aminotransferase; HbA1c: glycated hemoglobin; LDL: low-density lipoprotein; HDL: high-density lipoprotein

Test name	Result	Unit	Reference range
Complete blood count			
Hemoglobin	13.8	g/dL	13.0 - 17.0
RBC	4.64	10^6^/mm^3^	4.5 - 5.5
Total leucocyte count	5.37	10^3^/AuL	4 - 10
Platelet count	155	10^3^/AuL	150 - 410
Diabetes profile			
HbA1c	7.6	%	4 - 5.6
Glucose - fasting	168	mg/dL	70 - 99
Renal function test			
Blood urea nitrogen	7.98	mg/dL	09 - 23
Urea	22.08	mg/dl	19.26 - 49.22
Creatinine	0.88	mg/dL	0.70 - 1.30
Uric acid	4.6	mg/dL	3.5 - 7.2
Lipid profile			
Cholesterol - total	214	mg/dL	0 - 200
Cholesterol - HDL	42.8	mg/dL	≥40
Cholesterol - LDL	138	mg/dL	30 - 99.9
Liver function test			
Bilirubin - total	0.43	mg/dl	0.2 - 1.1
SGOT	20	U/L	0 - 34
SGPT	23	U/L	10 - 49
Alkaline phosphatase	61	U/L	46 - 116

## Discussion

Facial paralysis can result from lesions at various anatomical levels of the facial nerve pathway, and distinguishing between central and peripheral etiologies is critical for ensuring timely and appropriate management (Table 2).

**Table 2:** Differential causes of facial paralysis and key distinguishing features LMN: lower motor neuron; MRI: magnetic resonance imaging; DWI: diffusion-weighted imaging; CNS: central nervous system

Etiology	Anatomical level	Clinical pattern	Key distinguishing features
Bell’s palsy	Peripheral (facial nerve)	Acute unilateral LMN facial weakness	Retroauricular pain, altered taste, hyperacusis, absence of other focal neurological deficits
Supranuclear stroke	Central (corticobulbar pathway)	Contralateral lower facial weakness with forehead sparing	Preserved forehead wrinkling, associated limb weakness
Pontine infarct (facial nucleus/fascicle)	Brainstem (pons)	Ipsilateral complete LMN facial palsy	Sudden onset, vascular risk factors, associated brainstem symptoms, DWI restriction on MRI
Vestibular disorders	Peripheral vestibular system	Vertigo, imbalance	Positional triggers, absence of facial weakness or focal neurological deficit
Demyelinating disease	Central nervous system	Variable neurological deficits	Multifocal CNS involvement, characteristic MRI lesions
Neoplastic lesions	Central or peripheral	Progressive facial weakness	Gradual onset, mass lesion on imaging
Infective/inflammatory causes	Peripheral or central	Facial weakness ± systemic features	Fever, inflammatory markers, infectious history

Facial paralysis may be broadly classified into supranuclear (central), nuclear or fascicular brainstem, and peripheral causes. Supranuclear lesions typically spare the forehead, whereas pontine nuclear or fascicular lesions result in complete ipsilateral lower motor neuron weakness and may be accompanied by additional brainstem signs. Peripheral causes, such as Bell’s palsy, usually present without additional focal neurological deficits, and other less common etiologies can be differentiated based on associated clinical findings and imaging studies. In central lesions, such as those caused by supranuclear strokes, the paralysis predominantly involves the lower two-thirds of the face, sparing the forehead and eyelid due to bilateral cortical innervation of the upper facial muscles. This anatomical feature enables preserved forehead wrinkling and eye closure, as input from the contralateral motor cortex compensates for the damaged side [11]. In contrast, lesions involving the facial nerve nucleus or its fascicles in the pons disrupt both upper and lower facial innervation on the ipsilateral side, resulting in complete lower motor neuron facial palsy: a presentation that can closely mimic Bell’s palsy. Such pontine strokes are uncommon, accounting for approximately 1% of facial paralysis cases, but hold significant diagnostic and therapeutic implications due to their underlying vascular etiology [2,12].

Pontine infarctions involving the dorsal pons, particularly at the level of the facial colliculus, are typically associated with occlusion of small perforating arteries arising from the basilar artery. Risk factors such as hypertension, diabetes, and small vessel disease increase the likelihood of these lacunar strokes. Imaging plays a critical role in diagnosis; while initial CT scans may be unremarkable in early ischemic stroke, DWI is highly sensitive and usually demonstrates acute ischemic lesions within minutes to a few hours of onset. This underscores the need for high clinical suspicion and appropriate neuroimaging in patients presenting with isolated facial palsy who also have vascular risk factors.

Several case reports have highlighted this diagnostic challenge. In our patient, the lesion localized to the dorsal pontine facial colliculus, corresponding directly with the near-isolated lower motor neuron facial palsy. The presence of minimal additional neurological signs increased the risk of misdiagnosis as Bell’s palsy, emphasizing the importance of considering brainstem infarction even in presentations that appear predominantly peripheral. Agarwal et al. highlighted the need for careful radiological evaluation to differentiate pontine infarct from Bell’s palsy [6], while Novy et al. described a pure motor variant of pontine infarct manifesting solely as facial weakness [9]. Similarly, Agarwal et al. reported dorsal pontine lacunar infarcts presenting as isolated facial palsy, demonstrating the potential for misdiagnosis in the absence of detailed imaging [6].

This report emphasizes the importance of considering central causes, particularly pontine infarction, in patients presenting with facial palsy that appears peripheral, especially those with vascular comorbidities. In patients with unilateral facial paralysis accompanied by dizziness, headache, or other brainstem-related symptoms, Bell’s palsy should not be assumed to be the primary diagnosis without further evaluation. The presence of vascular risk factors and associated neurological features should prompt consideration of central etiologies, particularly pontine infarction, and guide appropriate neuroimaging to exclude brainstem pathology. Early recognition is crucial, as treatment with antiplatelet therapy and secondary stroke prevention differs significantly from the corticosteroid-based management of Bell’s palsy. Delays or misdiagnosis may prevent patients from receiving timely stroke interventions during the critical therapeutic window.

## Conclusions

This report demonstrates that pontine infarction can present as isolated lower motor neuron facial palsy, mimicking Bell’s palsy. Although this phenomenon is documented in the literature, our report highlights the importance of thorough clinical assessment in patients presenting with facial weakness that appears peripheral. Neuroimaging should be considered when facial paralysis occurs in the presence of vascular risk factors, atypical symptoms such as dizziness or dysarthria, or additional neurological findings. In these cases, a stroke-focused MRI protocol may be preferable to an IAC protocol to exclude a central ischemic cause.
